# Primary and secondary metastatic dissemination: multiple routes to cancer-related death

**DOI:** 10.1186/s12943-025-02389-5

**Published:** 2025-07-22

**Authors:** D. Sparrer, R. Blazquez, F. Keil, S. Einhell, F. Lüke, S. Uderhardt, C. Gerner, C.H.R. Wendl, M. Proescholdt, C. Schulz, A. Kandulski, S. Haferkamp, H.J. Schlitt, T. Bäuerle, K. Franze, R. Mayr, M. Rechenmacher, P. Hau, D. Hirsch, D. Heudobler, K. Evert, T. Pukrop

**Affiliations:** 1 Center for Translational Oncology, University Hospital Regensburg, Regensburg, 93053 Germany; 2 Bavarian Cancer Research Center (BZKF), Germany 93053 Regensburg,; 3 Department of Internal Medicine III, University Hospital Regensburg, Regensburg, 93053 Germany; 4 Institute of Pathology, University of Regensburg, Regensburg, 93053 Germany; 5 Department of Medicine 3 - Rheumatology and Immunology, University Hospital Erlangen and Friedrich-Alexander-Universität Erlangen-Nürnberg, Erlangen, 91054 Germany; 6 Deutsches Zentrum Immuntherapie (DZI), University Hospital Erlangen, Erlangen, 91054 Germany; 7 Department of Analytical Chemistry, Faculty of Chemistry, University of Vienna, Waehringer Str. 38, Vienna, 1090 Austria; 8 Joint Metabolome Facility, University of Vienna and Medical University of Vienna, Waehringer Str. 38, Vienna, 1090 Austria; 9 Institute of Radiology, University Hospital Regensburg, Regensburg, 93053 Germany; 10 Department of Neurosurgery, University Hospital Regensburg, Regensburg, 93053 Germany; 11 Department of Internal Medicine II, University Hospital Regensburg, Regensburg, 93053 Germany; 12 Department of Internal Medicine I, Gastroenterology, Hepatology, Endocrinology, Rheumatology and Infectious Diseases, University Hospital Regensburg, Regensburg, 93053 Germany; 13 Department of Dermatology, University Hospital Regensburg, Regensburg, 93053 Germany; 14 Department of Surgery, University Hospital Regensburg, Regensburg, 93053 Germany; 15 Preclinical Imaging Platform Erlangen, Institute of Radiology, University Hospital Erlangen and Friedrich-Alexander-Universität Erlangen-Nürnberg, Palmsanlage 5, Erlangen, 91054 Germany; 16 Department of Diagnostic and Interventional Radiology, University Medical Center of the Johannes Gutenberg-University Mainz, Langenbeckstrasse 1, Mainz, 55131 Germany; 17 Department of Physiology, Development and Neuroscience, University of Cambridge, Downing Street, Cambridge, CB2 3DY UK; 18 Institute of Medical Physics and Microtissue Engineering, Friedrich-Alexander-Universität Erlangen-Nürnberg, Erlangen, 91054 Germany; 19 Max-Planck-Zentrum für Physik und Medizin, Erlangen, 91054 Germany; 20 Department of Urology, Caritas St. Josef Hospital, University of Regensburg, Regensburg, 93053 Germany; 21 Center for Palliative Medicine, University Hospital Regensburg, Regensburg, 93053 Germany; 22 Department of Neurology and Wilhelm Sander Neuro-Oncology Unit, University Hospital Regensburg, Regensburg, 93053 Germany; 23 Fraunhofer Institute for Toxicology and Experimental Medicine ITEM-R, Regensburg, 93053 Germany

**Keywords:** Metastasis, Metastatic dissemination, Bio Therapeutic Goals of Cancer Care Model, Primary dissemination, Secondary dissemination, Cancer-directed therapy, CNS

## Abstract

Metastatic disease accounts for approximately 80% of cancer-related deaths, typically manifesting as single-organ failure mainly through abdominal, cardiovascular, neurological, or respiratory complications. Despite treating thousands of cancer patients daily worldwide, our understanding of organ-specific metastatic dissemination routes, tissue destruction mechanisms and reasons for organ failures remains limited. As cancer-directed therapies advance, maintaining organ function has emerged as a critical therapeutic goal of care. To develop more effective treatment strategies, a comprehensive understanding of the pathophysiology is essential, particularly regarding secondary and subsequent metastatic waves that lead to extensive macro-metastases and organ failure. Critical distinction between primary metastatic spread and secondary intra-organ dissemination is crucial. In the era of precision oncology, elucidating organ-specific destruction processes and the pathophysiology of metastatic waves is fundamental for advancing patient care. To highlight the emerging goal of care of maintaining organ function, we aligned the metastatic biology, clinical stages, goals of care and therapeutic indications: the Bio Therapeutic Goals of Cancer Care Model.

## Background/Introduction

In 2022, the estimated number of cancer deaths in the United States was 609,360. Among both women and men, the six predominant cancer types contributing to these mortality rates are colorectal, hepatic, pulmonary, pancreatic, ovarian, and prostatic cancers. Together, these cancer types account for approximately 310,420 deaths, representing 51% of all cancer-related fatalities [1]. It is important to note that not all of these cancer-related deaths are due to distant metastases, since these numbers also include patients with ovarian cancer who died from peritoneal metastases without any or only few distant metastases. Such cancer-related deaths are more likely due to primary intra-cavity than primary vascular dissemination. Furthermore, all aforementioned solid cancer types are known to utilize alternative routes of dissemination in addition to primary vascular dissemination. As early as 1972, a large case series investigating the dissemination routes of bowel cancer showed that at least three routes of metastatic dissemination are more or less equally detectable. Besides the primary vascular dissemination, this study highlighted per continuitatem (nearby metastasis) and intra-cavity dissemination routes. In the latter, the mesocolon, the mesentery of the small intestine, the peritoneal attachments of the ascending and descending colon clearly serve as watersheds that direct the flow of malignant cells [2]. Moreover, metastatic seeding in the peritoneal cavity follows gravity and flow, while slow-flowing areas or reservoirs are predisposed sites for seeding [3]. Further alternative (non-intravascular dissemination) routes are intra-luminal, perivascular and perineural spread. In this context, it is fundamental to emphasize that metastasis is not exclusively understood as vascular metastasis from the primary tumor by primary metastasis-initiating cells (primMIC). Rather, metastasis refers to all processes that ultimately lead to the formation of new metastatic tissue. Even the direct extension of a primary tumor into nearby tissues fulfills the definition of metastasis [4]. Thus, sometimes only body fluids, epithelial barriers or paper-thin connective tissue structures separate the metastatic source from the new metastatic host tissue. This simple clinical example illustrates that not all steps of the classic metastatic cascade are always required [5]. Nevertheless, schematic illustrations of the metastasis cascade are often simplified to five key steps: Invasion of the primary tumor, intravasation, survival in the vascular system, extravasation and colonization of a distant host organ. However, in the case of nearby metastasis, some of these steps (intravasation, survival in the vascular system, extravasation) are not mandatory at all.

Moreover, besides the number of anatomical barriers, a circulating tumor cell (CTC) must modulate the primary tumor microenvironment (TME), be mobile, invasive, reveal metabolic and mechanical plasticity, and perform intra- and extravasation. The successfully disseminated tumor cell (DTC) must again exhibit plasticity, metabolic rewiring, immune resistance and be able to colonize a distant organ, including the formation of a metastatic microenvironment (MME) [4, 6–12], a process which presumably is extremely ineffective and time-consuming. Most CTCs even fail directly after detaching from the primary tumor. However, primMIC that overcome all these hurdles gain special properties during the adaption to the new host microenvironment and reveal distinct molecular features. For example, brain metastases exhibit metabolic rewiring in comparison to the primary tumor cells [13]. In particular, the metabolic rewiring and the bypassing of the organ-specific immune defense during the metastatic outgrowth could accelerate recolonization and the formation of a new metastasis elsewhere in the same or other organ (secondary dissemination) [14]. Thus, already metastasized cells, which detach from the metastatic tissue (secMIC), no longer have to undergo all these adaptations. In this line, re-injection experiments of cells that have already successfully colonized an organ or tissue demonstrate that the recolonization of the same organ occurs more rapidly, indicating that secMICs are better equipped to colonize this specific host organ [15]. There is evidence of established metastases serving as a source for secMIC and secondary dissemination. Several in vivo experiments and clinical observations point to brain metastases as source of leptomeningeal seeding (recently reviewed in the paragraph: Direct and/or perivascular spread [16]). In vivo studies have also described the possible contribution of secondary dissemination from liver and lung metastases to the total metastatic burden [14, 17, 18]. Moreover, current transplantation trials of unresectable colorectal liver metastases provide compelling evidence supporting the concept of secMICs and secondary dissemination. Across the TOSO, TransMet, and SECA-I and II trials, fourteen patients (around 14 percent) developed new metastases in the transplanted liver [19, 20]. These trials included patients with controlled, yet unresectable, liver metastases, where the primary colorectal tumor had been removed months or even years prior to transplantation. This strongly suggests that the new metastases in the graft liver did not originate from primMIC but rather from circulating secMICs shed by existing liver metastases or from undetectable metastatic lesions in other organs (invisible phase of metastasis). Notably, slightly less recolonization was reported in the transplantation of liver metastases from neuroendocrine tumors [21]. Moreover, the liver transplantation seems to significantly influence the further course of the diseases. Considering the Milan criteria, a recent single-center study compared the relapse pattern after liver metastasis resection with a matched transplantation cohort. The result showed a completely different clinical picture. While after metastases resection progression occurred mainly in the liver, progression of distal lymph nodes and multifocal involvement dominated in the transplantation cohort [22].

However, if secMICs from well-controlled or clinically undetectable metastases (micro-metastases) from colon cancer and even neuroendocrine tumors have the potential to form a macro-metastasis within months, what is the potential of secMICs derived from rapidly progressing metastases? This idea alone raises numerous follow-up questions, such as does every metastasis successfully release secMICs and if not, what distinguishes these metastatic cells from the others?

These questions and clinical scenarios highlight the substantial gap in understanding metastatic dissemination between clinical practice and the existing literature. This review aims to bridge that gap by summarizing the different routes through which cancer cells spread to, within, and from metastatic organs and aims at shedding some light into the complex process of metastatic dissemination and organ destruction.

## Main text

### The plethora of cancer-related organ failures

Already in 1896, Friedrich Ernst Krukenberg (1871–1946) described the Krukenberg tumor. One alternative metastatic path to the ovaries is the above mentioned flow- and gravity-dependent direct dissemination of signet ring cells of gastric cancer from the primary gastric tumor to the ovaries in the abdominal cavity without entering the blood circulation (primary intra-cavity dissemination). Typically, signet ring cell carcinoma comes with poorly cohesive cancer cells that lose their epithelial function and infiltrate tissue with a diffuse histological growth pattern (HGP). This diffuse HGP is due to the loss or truncation of E-cadherin [23]. The loss of E-cadherin-mediated cell adhesion appears to be an important molecular feature for this special dissemination track, which potentially drives the detachment from the primary tumor and attachment to the ovaries.

**Fig. 1:**
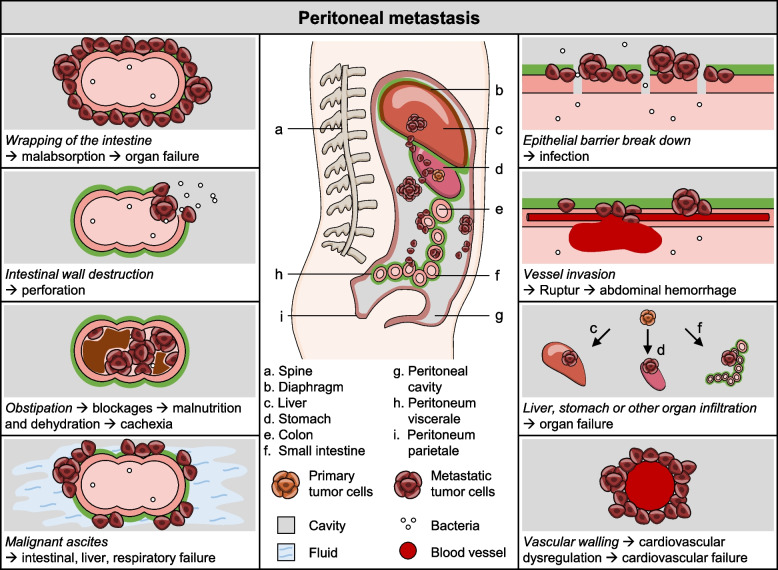
Peritoneal metastasis. Overview of local complications of peritoneal metastasis including wrapping of the intestine, intestinal wall destruction, obstipation, malignant ascites, epithelial barrier break down, vessel invasion, organ infiltration, and vascular walling

Similar to the Krukenberg tumor, primary intra-cavity dissemination is often the cause of peritoneal metastasis from other cancers of the gastro-intestinal tract. In this context, it is important to mention, that three of the most fatal cancers [24], namely colon, pancreas and ovary, have a high percentage of peritoneal metastasis in advanced tumor stages [25]. Peritoneal metastasis is accompanied by a poor prognosis and severe symptoms with a dramatic drop of quality of life (QoL). It interferes with intestinal functions and causes intestinal obstruction, which can subsequently lead to symptoms such as severe abdominal pain, bloating, constipation, malnutrition, vomiting and ascites. Ascites further causes abdominal distension followed by decreased lung expansion and subsequent hypoventilation mainly of the lung bases. Furthermore, peritoneal metastasis reduces the peritoneal lining and interrupts the epithelial barrier function for microbes (attenuation of the anatomical barrier), making it susceptible to peritonitis, septicemia and sepsis. Peritoneal metastasis can also impair circulatory regulation locally by surrounding (walling) the abdominal vessels to such an extent that it leads to cardio-vascular failure. Finally, peritoneal metastasis can serve as source for secMIC, which can infiltrate neighboring organs such as the liver, intestines, stomach and even the kidneys [25]. Theoretically, secMICs detached from the peritoneum could enter the lymphatic system via a specialized drainage system in the diaphragm, the lymphatic lacunae. This anatomical structure is composed of peritoneal stomata, lymphatic drainage units and subperitoneal terminal lymphatics, which absorbs fluids, particles and cells form the abdominal cavity [26]. Overall, secMICs detaching from established peritoneal metastases are a potential source of metastasis to intra-abdominal organs, such as the liver, intestine, stomach and nearby tissues (e.g. pleura, kidneys) (Fig. 1). Why is this clinically relevant? A high percentage of patients with ovarian and colon carcinoma most frequently die from local complications of peritoneal metastasis [27, 28]. At least in these cases the primMIC do not have to enter the blood circulation to cause organ failure and death.

In contrast, if the cause of death is a massive tumor embolism derived from the primary tumor, then tumor embolization can lead to a rapid organ-failure without ever having successfully completed the final step of metastasis, the colonization of a new host organ. This rare clinical scenario appears to occur particularly in primary tumors with vascular invasion and intravascular growth [29, 30]. A further scenario are lung cancer and melanoma, which are more likely to metastasize to distant organs [31, 32]. Patients with late stage malignant melanoma almost exclusively die from the consequences of vascular metastasis with multiple metastases in various organs. Most patients succumb to respiratory failure or central nervous system (CNS) failure caused by the metastatic disease in the respective organ. Neurological death due to CNS metastasis is also very often seen in driver-associated non-small cell lung cancer (NSCLC) [33]. A special situation is uveal melanoma, which typically leads to liver failure caused by multiple liver metastases that can occur even years after diagnosis.

**Fig. 2:**
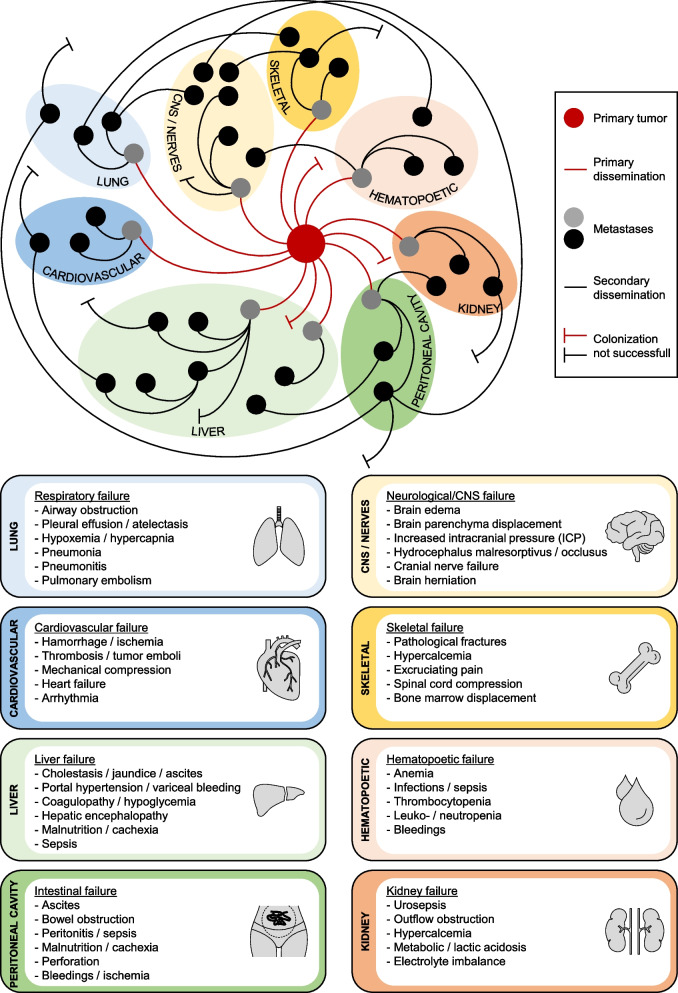
Overview of potential life-threatening scenarios and respective organ or compartment failures

However, regardless of the dissemination tracks, most cancer patients ultimately die from clinical complications related to the organ or compartment most severely affected. However, before patients die, they go through several therapies and waves of metastasis or local recurrence/progression. This in turn results in very individual patient journeys, organ-specific complications with related quality of life (QoL) changes [34] and various cancer-related causes of death (Fig. 2). Overall, all these clinical examples clearly illustrate the wide variability in patterns of organ failure, its impact on QoL and on the individual cause of death.

**Fig. 3:**
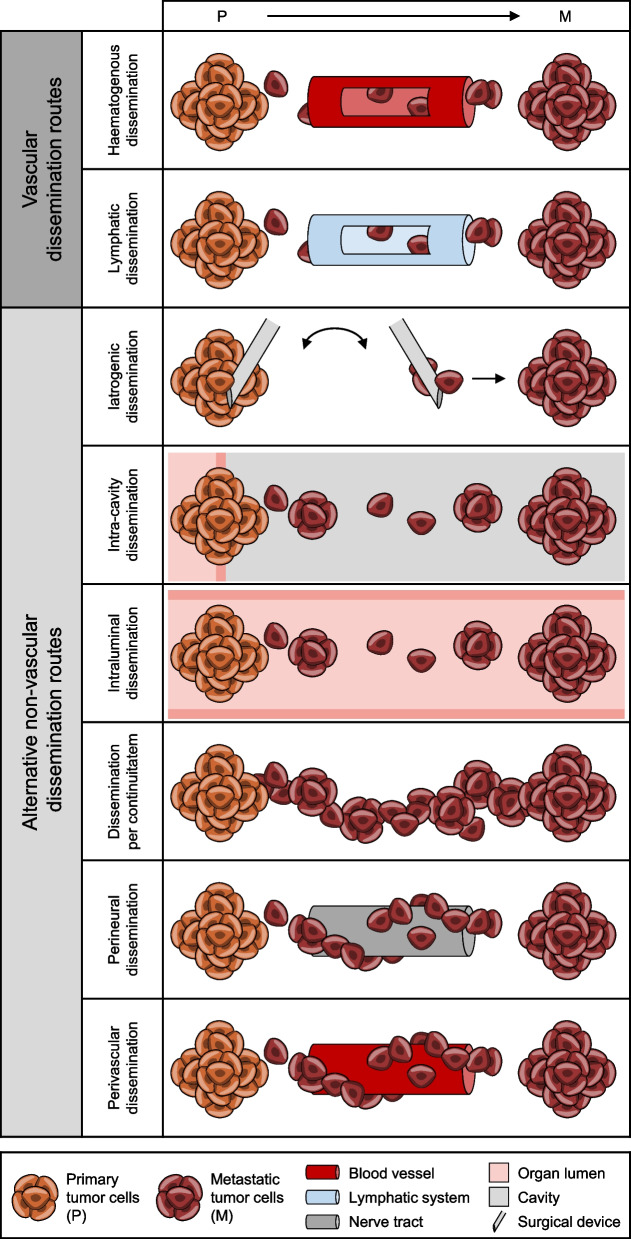
Overview of vascular and alternative non-vascular dissemination routes. Vascular dissemination routes include haematogenous dissemination and lymphatic dissemination. Alternative non-vascular dissemination routes include iatrogenic dissemination, intra-cavity dissemination, intraluminal dissemination, dissemination per continuitatem, perineural dissemination, and perivascular dissemination

In order to process these complex topics and clinical experience in a structured manner and to translate the biology into clinical decision-making, we continue with a detailed overview on the different dissemination routes, explain their consequences and finally convert them into a biology-driven clinically relevant model. For this, we have structured the subsequent chapters as follows:

- primary dissemination: the various routes of dissemination from the primary tumor
- secondary dissemination: the dissemination from metastatic tissue and recolonization
- factors influencing the different dissemination routes: space, flow-speed, filter organs, barriers and defence mechanisms
- the Bio Therapeutic Goals of Cancer Care Model: translation of the metastatic biology and stages into goals of care and therapeutic indications

### Primary Dissemination

Regardless of how the patient journey of a metastatic patient ends, it always begins with tumor initiation and primary dissemination. The timing of primary dissemination is certainly variable, but as we know, it can occur very early after tumor initiation and long before the primary tumor is diagnosed, during the invisible or cryptic phase of primary tumor growth [35]. Model calculations of patients with non-small cell lung cancer from the TracerX cohort confirm this statement. These calculations show that primary metastatic dissemination is more likely to occur when the tumor diameter is less than 8 mm, which is the current threshold for further investigation of incidentally detected peripheral lung lesions [36].

### Primary vascular (haematogenous/lymphatic) dissemination

For years, classical primary vascular dissemination, often involving traversal through the lymphatic system, has served as the foundational model for studying the five key steps of metastasis, and it has been intensively reviewed. Current reviews deal with a range of related topics including CTCs, cell clusters versus single cells, homotypic versus heterotypic clusters [37–40], fluids and their mechanics [41], epithelial mesenchymal transition (EMT) [42, 43], mechanotransduction [44], the concept of epithelial mesenchymal plasticity (EMP) [45, 46], cancer cell metabolism [7, 8, 47] and metastasis-initiating cells (MICs) [6]. In this review, we will address some general aspects and focus on primary alternative and secondary metastatic dissemination routes of carcinomas.

Primary vascular dissemination is typically characterized by long-distance spread, allowing CTCs to travel directly to distant sites without intermediate stops. This characteristic depends on the frequent need of CTCs to pass filter-organs (e.g. liver, lung) [48], as well as the limited space and high shear forces in the capillary system. Moreover, CTCs have to sustain high flow speed and pulsatile turbulent flow in the arteries [41]. The observations of Cole and colleagues in 1958 already indicated this, when they found large primary tumor clumps in the central draining veins of many tumor patients of colon cancer, but only single cells in the peripheral blood, if at all [48]. They concluded that this is due to the passage of CTCs through two filter organs, the liver and the lungs. This assumption is still valid today and continues to explain the typically low number of CTCs [48] detected in colon cancer.

The finding of large primary tumor clumps in the central draining veins was nevertheless the starting point of the no-touch isolation technique for primary tumor resection [49, 50]. In the standard technique, the tumor-bearing colon segment is mobilized first, followed by central vascular ligation (CVL). In contrast, the no-touch technique performs the CVL first, followed by the mobilization of the tumor-bearing colon segment, aiming at reducing the risk of vascular dissemination. However, the results of a phase III trial revealed no significant differences between these two surgical approaches [51]. The biology of the tumor clumps may help explain why no significant difference was observed. Experimental data revealed that when colon cancer cells are separated from each other, they lose the protection conferred by their cancer cell neighbors and perish. Indeed, cell aggregate formation, especially the cell–cell contacts, confers resistance to anoikis (*homelessness*) [37, 38, 52], even of benign gastro-intestinal cells [53]. In addition to the potential benefits of cancer cell aggregate formations, the bloodstream contains numerous other cells and particles that contribute to an intensive crosstalk [54], including the formation of neutrophil extracellular traps (NETs). These net-like structures composed of DNA, histones, and various proteins have a defensive function and represent very potent threats to combat [38, 55]. Nevertheless, they can also support the formation of clots with MICs [56–58] and thus promote their extravasation [59]. Overall, there are many dangers and obstacles lurking on the road to distant organs, meaning that only a small fraction of primMICs, if any, will successfully reach their target.

However, knowing for decades that bowel cancer disseminates in a high percentage also via intra-cavity or per continuitatem and that potential primMICs disseminate along the anatomic planes, the current state of the art provides the complete mesocolic and mesorectal excision in colorectal cancers. This includes not only the precise removal of the bowel segment but also the surrounding meso, with all the blood and lymphoid vessels, lymph nodes and fat contained therein. These surgical standards emphasize the careful dissection along anatomical planes to achieve clear margins and complete removal of nearby hidden MICs. These techniques led not only to a reduction of local relapses, but also to an improvement in overall survival [60, 61]. The number of containing lymph nodes in the mesocolic/mesorectal directly correlates with prognosis, even in UICC Stadium II [62]. The surgical en bloc specimen should contain a minimum of 12 lymph nodes or more to determine a pN0 status; and therefore considered for the indication of potential adjuvant treatment [63]. Recently, the DCGC01 study raised the same question in gastric cancer, whether a complete mesogastric excision would improve survival [64]. Also in breast-conserving surgery in stage I-III breast cancer, the technique of cavity shave margins significantly reduces local recurrence and has thus prevailed over the years [65–69]. These clinical examples emphasize the importance of alternative primary dissemination routes, which have been taken into account in surgical techniques for decades. Most importantly, this surgical approach saved the lives of thousands of patients.

### Alternative primary non-vascular dissemination routes

The peritoneal cavity (intra-cavity dissemination), airspaces [70] or intestinal lumen (intra-luminal dissemination), provide almost unlimited space for primMICs and the direction of dissemination in these spaces follows the gravity and flow. Moreover, in these scenarios the primMICs do not have to cross filter organs or sustain high shear stress as well as fast and turbulent flow-speeds. Thus, they are more likely to remain structurally intact including the preservation of their cell–cell contacts. Indeed, colon cancer clusters are more effective in the development of peritoneal metastasis [71, 72]. In addition to all the obstacles and dangers of vascular dissemination missing during intra-cavity dissemination, large tumor clumps also have a time advantage over single CTCs because they start colonization with a much higher tumor cell count. This may also influence the tumor volume doubling time (TVDT) [35] and potentially shorten the invisible or cryptic phase of metastasis [6, 35].

The example of alternative dissemination tracks of colon cancer illustrates the clinical significance and the necessary adjustments to clinical (surgical) procedures. With this in mind, we will take a closer look at some of these potentially clinically relevant alternative or complex dissemination routes, which are summarized in Fig. 3.

#### Iatrogenic dissemination and the role of the resection cavity

Since the early days of visceral surgery, local recurrence has been associated with wound healing after surgery [73], and surgeons have been aware of the risk of metastatic peritoneal implants from needle sticks [48]. Previous studies have also shown that intraluminal exfoliated cancer cells (ECCs) [74] can also disseminate and form metastases in the intestine. This mechanism is suspected to be a potential source of local recurrence within the first two years after surgery, particularly in rectal cancer. Currently, the tumor-bearing colon is resected at least 10 cm away from the tumor tissue in both directions, independent of the surgical strategy (extent of resection [75–79] and timing of resection [49, 50]) and meticulous care is also taken to ensure that no devices are used during suturing that have previously touched the primary tumor. In particular, in rectal cancer intraoperative irrigation (intraluminal washouts) are additionally performed to reduce the number of ECCs in the intestine[80]. For more than 100 years now, the surgical technique has been refined in order to take into account the biological and iatrogenic alternative dissemination pathways. Now, all current surgical techniques take nearby hidden MIC into account to reduce iatrogenic tumor cell dissemination to a minimum.

#### Primary intra-cavity dissemination

Intra-cavity dissemination is a specific route where cancer cells directly shed from a primary tumor into cavities, and can subsequently attach onto the neighboring surfaces. As described above, this applies to the dissemination of ovarian, colon, pancreatic and gastric cancer cells into the abdominal cavity. Lung carcinoma, the primary tumor with the highest rates of cancer-related deaths, also uses the intra-cavity dissemination path in the pleural space. Primary tumors of the brain also use the cerebrospinal fluid (CSF) space as dissemination track. Additionally, these cavities are also used by secMICs during secondary dissemination (see below). Remarkably, intra-cavity dissemination has also been described for benign metastasizing leiomyoma [81], underlying the previous acknowledgement that primary dissemination not always requires tumor progression [82].

#### Primary dissemination per continuitatem

Metastasis via dissemination per continuitatem refers to the direct spread of primary tumor cells by extension from one tissue or organ to a nearby anatomical structure or host organ as described above. Besides the cancer types of the GI tract, lung cancer also often growths directly into nearby structures, as the chest wall, spine or the anatomical structures (e.g. blood vessels) of the mediastinum. A prime example is the so-called Pancoast tumor, typically an adenocarcinoma located at the apex of the lungs where the tumor invades the adjacent anatomical structures, such as the first ribs, the vertebral bodies, the brachial plexus, the phrenic nerve, the sympathetic trunk, the recurrent laryngeal nerve and subclavian vein and artery. Pancoast tumors account for approximately 5% of all lung cancers [83].

#### Primary perineural dissemination (PND)

At the latest since 1835, it is known that tumor cells from a primary tumor can grow perineurally, and since 1935, it is described that they can disseminate along the nerve tracts [84–86]. In the case of perineural dissemination (PND), Schwann cells come into direct contact with primary tumor cells leading to the formation of Schwann cell-cancer cell interactions, which seems to promote tumor cell protrusions and detachment from the primary tumor. After detachment, the nerves serve as anatomical guiding rails for the primMICs, which are located on the surface or even inside the nerve sheet [86–90]. This observation also led to the definition of four different types of perineural invasion (PNI): PNI 0 = no tumor cells around the nerve sheaths, PNI 1 = tumor cells surround nerves less than 33%, PNI 2 = tumor cells encircle at least 33% and PNI 4 = tumor cells infiltrate the nerve sheaths. The incidence of PNI ≥ 1 is up to 98% in pancreatic tumors, 80% in head and neck, 75% in prostate cancer and cholangiocarcinoma, and 33% in colorectal cancers [87]. Clinically, positivity for PND is often associated with poor outcome [91–100] and has therefore been included in the TNM classification. Most importantly, detection of PNI in the transrectal ultrasound-guided biopsy specimen is a powerful predictive histopathological feature for bone metastasis of prostate cancer [101].

Given the interactions between Schwann cell and cancer cell interactions as a prerequisite, it is not surprising that cell adhesion and surface molecules have been identified in the context of PND. For example, L1 cell adhesion molecule (L1CAM), initially linked to neuronal migration, is overexpressed in pancreatic cancer PNI. Neural cell adhesion molecule (NCAM), expressed on neurons and Schwann cells, also facilitates PNI by promoting cancer cell dissemination. MUC1, found on pancreatic cancer cells, interacts with Myelin-associated glycoprotein (MAG) on Schwann cells, enhancing cancer cell survival within nerves [102–104]. The exact mechanisms underlying this dissemination process remain barely understood. However, as with other alternative dissemination routes, specific cell adhesion and cell surface proteins appear to play a crucial role.

### Dissemination routes into or via the CNS compartment

#### Dissemination route of primary leptomeningeal metastasis (LMM)

Leptomeningeal metastasis (LMM) refers to the appearance of metastatic cells in the leptomeninges (pia mater and arachnoid mater) and is often accompanied by the detection of cancer cells in the cytology of the CSF. LMM in carcinomas is also described as carcinomatous meningitis, meningeal carcinomatosis or neoplastic meningitis, which also includes leukemic meningitis (in leukemia) and lymphomatous meningitis (in lymphoma).

Once in the CSF, metastatic cells can further disseminate along the meningeal surfaces or follow the CSF-flow. Comparable to the abdominal cavity, predilection sites of colonization are regions with slow flow and/or gravity-dependent locations [105, 106] and potentially anatomical structures where CSF resorption or drainage takes place. Besides typical signs of intracranial pressure (e.g. headache, mental changes, clouding of consciousness, nausea, and vomiting) LMM could present with a plethora of further symptoms such as gait difficulties, cranial nerve palsies with diplopia, visual disturbances, and/or hearing disorders, indicating a direct nerve affection. If these unusual symptoms occur in patients with breast cancer, lung cancer or malignant melanoma without other symptoms, one should consider LMM and have a diagnostic clarification carried out if further suspicion arises [105].

The potential routes of dissemination to the meninges have recently been reviewed [16, 107]. Nevertheless, we will briefly discuss the question how MICs may colonize the cranial nerves as first site of metastasis. If this very seldom metastatic event of breast cancer, lung cancer and malignant melanoma is caused by primMICs, a series of complex and sequential steps – combining primary vascular and alternative dissemination routes – must be successfully completed. This hypothetical cascade includes: (1) vascular dissemination of primMICs; (2) primMIC cross the blood CSF barrier at the choroid plexus (e.g. at the Bochdalek´s flower basket) [108] or via the arachnoid-CSF barrier [16]; (3) flow dependent further dissemination of primMIC via the CSF [105]; (4) gravity- and flow speed-dependent attachment of primMICs at the meninges [105]; (5) primMICs attach at the nerve sheaths of the cranial nerves. This may be along the perivenous space at the CSF resorption points, analogous to the CSF efflux route [109]. Then (6) the cells again spread along the cranial nerves and (7) finally colonize them. Interestingly, the affected nerves are mainly nearby the CSF cisterns, like the chiasmatic or pontine cistern, which are in CSF slow-flow areas and in direct proximity to the Bochdalek´s flower basket. These observations thus support the plexus as potential entry and the hypothetical sequence described.

#### Perineural dissemination as a source of isolated cranial nerve or meningeal metastasis

A completely less complex explanation for metastasis to the cranial nerves would be perineural dissemination. As opposed to colonization of the cranial nerves as the first sign of LMM, the cranial and spinal nerves serve retrograde dissemination tracts into the CSF-space. Thus, primMICs or secMICs can travel along spinal or cranial nerves to reach the leptomeninges. This route may be conceivable for primary tumors nearby the CNS like head and neck cancers or prostate cancers that have a propensity for PND. However, these cancer types develop very seldom LMM or isolated cranial nerve colonization and in the case of breast cancer, lung cancer or malignant melanoma this route is rather unlikely.

#### Primary and secondary dissemination per continuitatem to the meninges

In contrast to PND, head and neck cancers are more often known to directly metastasize through the skull base (dissemination per continuitatem of primary tumors) into the CNS, naturally affecting the dura mater first. Also, other primary tumors in proximity to the brain or spinal cord, such as vertebrae or paraspinal structures, can disseminate directly into the dura mater and subsequently the leptomeninges. However, the most common case of dissemination to the meninges by continuous spread are bone metastases of the spinal column and sometimes bone metastases of the skull (secondary dissemination per continuitatem metastases). Moreover, the direct connection to the CSF space via the nasopharyngeal lymphatic plexus [110] and the skull channels [111] open up previously completely unnoticed pathways of dissemination of the spine and skull into the CSF space and brain parenchyma.

#### Dissemination on the surface of the emissary vessels or ‘skull channels’

**Fig. 4:**
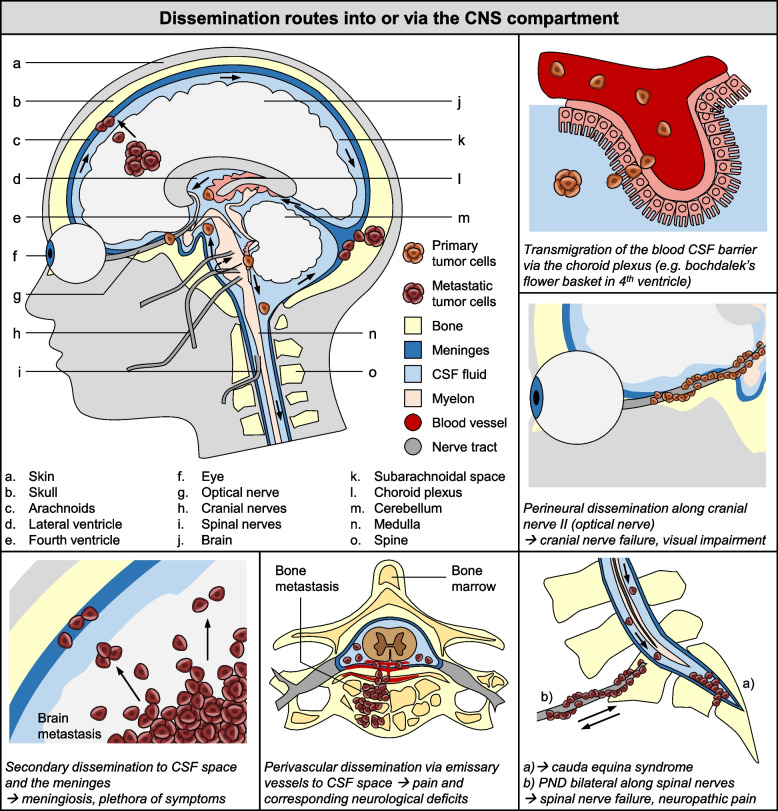
Overview of dissemination routes into or via the CNS compartment. Dissemination routes include transmigration of the blood CSF barrier via the choroid plexus, perineural dissemination along cranial nerve II (optical nerve), perineural dissemination bilateral along spinal nerves, perivascular dissemination via emissary vessels to CSF space, and secondary dissemination to CSF space and the meninges. CSF: cerebrospinal fluid, PND: perineural dissemination

The discovery of channels in the skull and vertebral bone, the so called ‘skull channels’, connecting the bone marrow directly with the meninges has revealed not only new immune gateways to the CNS but also completely unexpected dissemination routes of MIC to the meninges and finally CNS [111]. Yao and colleges have already described a direct dissemination route from the bone marrow via the emissary vessels (perivascular) into the CSF space in acute lymphatic leukemia (ALL) [112]. Similar to ALL, this dissemination route could theoretically also occur via DTCs of the bone marrow. However, bone metastases and bone marrow carcinoma metastasis are more likely to follow this dissemination route. However, in this case it is clearly a secondary dissemination track. The different dissemination routes into or via the CNS compartment are described in Fig. 4.

### Secondary Dissemination

‘Do metastases metastasize?’ is a long-standing question in metastasis research [113, 114]. However, the question was mainly raised with regard to metastatic dissemination to other organs and to a lesser extent in the context of further spread of metastatic cells within the same organ and its subsequent destruction. Moreover, mainly sequential considerations and calculations were made, but they overlooked the fact that secondary dissemination can also start in parallel to the formation of the first micro-metastasis (invisible time of metastasis). If cells from micro-metastases would also disseminate as early as the primary tumor, the timespan between the first and subsequent metastasis in the same organ will be undoubtedly short. Moreover, since the cells have already colonized this organ, the secMIC are well prepared to recolonize the same organ. The recolonization of the graft liver by colon or neuroendocrine metastases in the transplantation trials described above at least provide evidence that a few months are sufficient to recolonize an organ.

Nevertheless, can we be sure that secMICs can actually also disseminate from metastatic tissue and contribute to disease progression? Similar to the peritoneal implants of primary tumor cells by needle stiches, metastatic cells detached from the metastatic tissue during resection are also a source of iatrogenic seeds. This scenario is well described for sarcoma. To prevent iatrogenic dissemination the clinical guidelines even recommend core needle biopsies and to avoid incision biopsies. Moreover, the pre-treatment biopsy have to be performed in such a way that the biopsy tract can be safely removed at the time of definitive surgery [115]. This procedure is implemented in all clinical sarcoma guidelines and applies to resectable situations including a resectable metastasis. Actually, one should ask the other way around: Why should only primary tumors be able to disseminate and not metastases as well? What evidence is there to support this hypothesis?

### Pachymenigeal Disease (PMD)

Even in the early days of brain metastasis resection, the neurosurgeons were aware that piecemeal resection leads to higher rates of meningeal seeding and subsequent meningeal metastasis than en bloc resections. Comparable to incision biopsy of sarcomas, piecemeal resection of a brain metastasis is a risk factor for the dissemination of secMICs. Most recently, meningeal metastases have been studied in more detail and a new type, pachymeningeal disease (PMD), has been described in the clinics. Typically for PMD is that it can occur after resection and radiotherapy. Moreover, PMD significantly differs from LMM in appearance and prognosis. While classical LMM typically appears along the cerebellar folia and supratentorial sulci with a characteristic linear (sugar-coating) contrast enhancement on MRI, PMD exhibits nodular enhancements of the meninges, located outside of the respective radiotherapy target volume [116, 117]. In this context, it is striking that one of the multivariate tested factors is the extracranial controlled tumor status because PMD occurs typically months after resection, on average after 9 months. Of course, this time span is comparable to the recolonization of the graft livers described above and thus sufficient that metastatic tumor clumps colonize also the meninges. Moreover, it also demonstrates that patients are still alive months after diagnosis of brain metastases and goes for follow-up imaging. This is certainly due to the better extracranial control of the improved systemic therapy in the last decades. However, this also requires a potential adjustment of the sequence and procedures of local therapy strategies. For example, initial trials are attempting to reduce the risk of PMD through neo-adjuvant radiotherapy before resection of brain metastases.

It is also interesting to note that not all brain metastases cause PMD. This raises the question of which molecular mechanisms could play a role in PMD. A key biological distinction lies in the growth patterns of primary tumors versus macro-metastases. While all primary tumors of carcinomas are invasive and form an invasive front, their corresponding brain metastases can also show a non-infiltrative growth pattern at the macro-metastasis organ parenchyma interface (MMPI) [118–120]. Moreover, non-infiltrative metastases have a much better prognosis. This phenomenon is not only observed in brain metastases [118], but is most frequently and extensively described in liver metastases of colon cancer [121–123]. Most important, very recently we were able to demonstrate in a prospective image-guided neurosurgical feasibility trial that 0% of non-infiltrative metastases did develop meningeal metastasis, while 27% of diffuse infiltrative metastases did (manuscript submitted). This result indicates a potential connection of the HGP at the MMPI and the development of PMD. However, mechanistic studies to explain the different HGPs are almost missing and this topic receives little or no attention in translational research. In order to further develop pathobiology driven therapeutic strategies for brain metastases, these crucial questions need to be answered.

### Secondary intra-organ dissemination and intra-organ distant re-start of colonization

Besides the above-mentioned PMD, secondary dissemination of brain metastases could also occur per continuitatem, without any surgery. We described these intra-organ dissemination tracks for brain, liver and lung in [120].

Taken together, secMICs can perform [1] secondary iatrogenic dissemination (during surgery), [2] secondary dissemination per continuitatem, and [3] secondary intra-organ dissemination mainly based on existing anatomical structures [120]. Additionally, [4] secondary metastatic dissemination may also occur via anatomical channels to other tissues, like the skull channels [111] and [5] even secondary vascular dissemination also via special anatomical access routes as described above for peritoneal metastasis. However, in contrast to primary dissemination, in particular primary vascular dissemination, the processes, mechanisms, and genetic and molecular predispositions underlying secondary dissemination within the affected organs remain largely unknown.

### Factors influencing the different dissemination routes of primMICs and secMICs

The microenvironment of different body fluids significantly influences the phenotype and adaptability of metastasis-initiating cells [124]. Peritoneal fluid exhibits distinct characteristics in flow dynamics, intracavity pressure, and molecular and cellular composition compared to blood vessels. Similarly, the CSF system, accessed by tumor cells through the choroid plexus, presents a unique microenvironment. The seeding mechanisms of tumor cells in the target organ also differ fundamentally: while CTCs primarily arrest in capillaries during classical haematogenous dissemination, MICs in the peritoneal cavity or CSF system must adhere to epithelial surfaces such as the meninges or serous membranes of the peritoneal and pleural cavities. This distinct requirement emphasizes the critical role of cell adhesion mechanisms in these alternative dissemination routes [125]. Table 1 provides a comprehensive comparison of these primary dissemination pathways.

**Table 1:** Characteristics of vascular and alternative dissemination routes

Dissemination route	Vascular	Per continuitatem	Intra-cavity	Perineural	Complex (LMM)
**Space and distance**					
Distance	long	short	medium	short/medium	long
Space	limited space in the capillaries of the affected organ	immediate neighborhood	almost unlimited space	along anatomical tracks	limited space in the capillaries of the plexus choroideus
**Intra-vascular/cavity conditions**					
Flow-speed	fast	not applicable	slow	not applicable	different flows; the site of colonization seems flow- and gravity-dependent
Shear forces	high	none	low	none	different shear forces
Flow-type at the most extreme point during metastasis before colonization	turbulent	not applicable	laminar	not applicable	turbulent
(Blood-) Pressure	high	different pressures	low	not applicable	different pressures
Intravascular defense systems	contact with all defense systems in the blood	depends on localization	contact with the peritoneal or pleural defense systems	unclear	contact with intravascular and CSF defense systems
**Passage of filter organs and other barriers**					
Filter organs	lung, (liver)	not applicable	not applicable	not applicable	lung, (liver)
Additional barriers	depends on the target organ (e.g. blood–brain-barrier)	anatomical properties of the nearby target (mesothel, bone)	mesothel	unclear	blood-CSF-barrier
**Colonization of the Organ**					
Entering	from the inside via organ capillaries	from the outside	from the outside	unclear	from the inside via the plexus choroideus
Defense system during first contact	RTMs, BAMs	RTMs, BAMs	RTMs, BAMs	unclear	RTMs, BAMs
Defense during early colonization	RTMs, OSMs	RTMs, OSMs	RTMs, BAMs	unclear	RTMs, BAMs
**Metastatic cascade inclusive cause of death (COD)**					
Minimal set of metastatic key steps	5/5	1/5 (= colonization) + direct extension into the affected organ	1/5 (= colonization) + dissemination via body fluids	1/5 (= colonization) + dissemination via nerves	5/5
Special characteristics	all hallmarks of cancer are affected	unclear	cell adhesion molecules (E-cadherin)	cell adhesion molecules	cell adhesion molecules
Adaption to special fluids before start of colonization	blood	not applicable	body fluids in the pleural or abdominal cavity	not applicable	blood and CSF
Potential cause of death (COD)	organ failure of one (localized organ failure) versus multiple organs (systemic organ failure)	depends on the affected organ	failure of intra-cavity organ(s) (e.g. lung, gut), circulation or barrier break down, secondary dissemination (e.g. liver)	mixed clinical picture often with multiple bone metastases	CNS failure

*BAM* Border-associated macrophage, *CNS* Central nervous system, *CSF* Cerebrospinal fluid, *OSM* Organ-specific macrophage, *RTM* Resident tissue macrophage

Regardless of the metastatic route, early colonizing cancer cells invariably encounter spatially well-organized resident tissue macrophages (RTMs) as their first line of defense [126]. While RTMs interact with primMICs through various defense mechanisms, including growth suppression and dormancy induction [127], the molecular details of these interactions remain poorly characterized, particularly along alternative dissemination pathways. The specific responses of specialized tissue macrophages—including serosal macrophages to adherent colon carcinoma cells, pleural macrophages to lung carcinoma cells, and border-associated macrophages (BAMs) of the meninges to cerebrospinal fluid-borne tumor cells—remain largely unexplored. Although the interactions between primMICs and Kupffer cells [128] in the liver or microglia in brain tissue [126] are better understood, the responses of alveolar macrophages, osteoclasts, and other phagocytic cells to metastatic invasion require further investigation [129]. Understanding these frontier defense mechanisms, particularly the MIC-BAM and MIC-RTM interactions at tissue borders and interfaces, is crucial for developing effective strategies against metastases.

The formation in which MICs detach from primary tumors represents a crucial factor across different dissemination routes [124]. Since tumor progression and EMT are no longer prerequisites for metastasis [82], even large, well-differentiated tumor clumps can initiate metastases. The peritoneal cavity particularly accommodates the transport of substantial tumor aggregates. These aggregates may differ not only in cell number and tumor volume doubling time (TVDT), but also in their fundamental properties. Certainly, the preservation of the epithelial structure during dissemination has different molecular purposes than the E-cadherin lost in cells of a Krukenberg metastasis. These enormous differences in the cell adhesion molecules suggest that the alternative dissemination routes differ from one another not only histologically but also on a functional and molecular level. Moreover, large tumor clumps can comprise hundreds of well-differentiated MICs maintaining their epithelial phenotype, complete with functional barrier formation, mucous production, and associated microbiome. This contrasts sharply with single, metabolically reprogrammed carcinoma cells displaying mesenchymal features, which cannot establish epithelial barriers.

These distinctions could significantly impact the TVDT [35], MIC-BAM and MIC-RTM interactions, immune system responses, histological growth patterns, immune infiltration patterns, and metastatic matrix composition. The type of metastatic colonization may be influenced by these characteristics. At least two distinct histological growth patterns (HGPs) have already been identified during early colonization of vascular-disseminated MICs: perivascular and spheroidal [130]. These patterns influence both the initial immune response and subsequent metastatic progression. Furthermore, the HGP at the MMPI significantly affects prognosis and therapeutic outcome [120].

#### The Bio Therapeutic Goals of Cancer Care Model and Future Directions

**Fig. 5:**
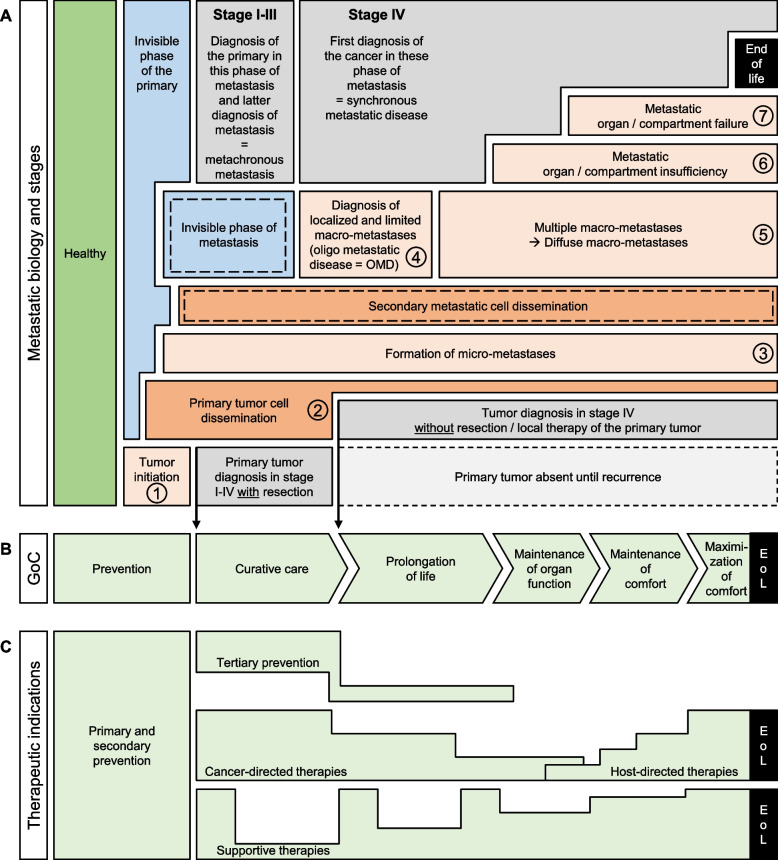
Bio Therapeutic Goals of Cancer Care Model. Interplay of (**A**) metastatic biology and stages, (**B**) goals of care, and (**C**) therapeutic indications. EoL: end of life, OMD: oligo metastatic disease

All the clinical scenarios and in vivo experiments mentioned above at least indicate that primMICs and secMICs could exist in parallel in different phases of metastasis in different organs and compartments. However, this aspect as well as the second and later waves of metastasis, the metastatic organ destruction and tumor-related mortality, are not part of most illustrations and descriptions of the metastatic cascade. These illustrations often end with the development of the first organ metastasis and avoid portraying the final and life-threatening processes and cause of death (Fig. 5A, see phase 5 to end of life). Nevertheless, we perform many cancer-directed treatments in these phases of metastasis without understanding the underlying biology. Due to the responsibility we also have for late-stage and heavily pre-treated tumor patients, we must not exclude these phases from our considerations and scientific efforts. For example, improved knowledge about the obvious biological differences of liver macro-metastases could improve the selection of patients for resection or even liver transplantation. It is quite conceivable that liver metastases that show almost no detachment of secMIC or secondary dissemination could be favored for resection or even liver transplantation. This biological stratification could also apply to other situations with oligo-metastatic disease (OMD). Recently there was a comment, which underlines the missing biomarkers and significance of the biological features in the OMD situation [131]. Now, we only have a clinical snapshot for decision-making in the OMD situation, which does not tell whether there is limited metastasis over time or whether we have diagnosed the tip of the metastatic iceberg. This clinical scenario concerns precisely the transition between limited- and multiple macro-metastasis (Fig. 5A, transition of phase 4 to 5). Moreover, we are now exploring more and more inexplicable and unexpected treatment responses even in seemingly hopeless patient journeys (Fig. 5A, cancer-directed treatments in phase 6 or 7) [132]. Even in patients with NSCLC and multiple brain metastases or meningeal metastases with driver alterations, prolongation of life would clearly be the Goal of Care (GoC) (Fig. 5B). In this context, a recent study with NSCLC patients and brain metastasis reported that the median time to intracranial progression was not reached after 60.2 months of follow-up and the probability of being free of intracranial progression was 83% after 5 years [133].

However, even in the era of targeted therapies and personalized medicine we still use simple clinical markers also in these clinical late stage scenarios to assess the prognosis for treatment decisions and therapy monitoring. These parameters include the number of metastasized organs, the number of detectable metastases, synchronous or metachronous metastases, localization of metastases, previous therapies and, of course, the patient's fitness [28, 134, 135]. Randomized systemic therapy studies or innovative biomarker trials hardly exist for patients with CNS metastasis or heavily pretreated patients. The search of potential biomarkers representing the pathophysiology of the later phases of metastasis, such as the transition from limited to multiple macro-metastasis, the status of organ destruction and finally hinds to the potential cause of death, are rare (Fig. 5A, transitions of phase 4 to 8). One of the few studies described in some cases with NSCLC brain metastases weeks before cancer-related death a significant increase of lipocalin-2 [136]. However, the missing clinical trials or systematic research in this area could explain why we are still unable to predict the individual response in different compartments to different cancer-directed therapies during these phases of metastasis. As a result, many patients still receive cancer-directed therapies up to 30 days before their death. However, these ineffective therapies not only cause undesirable side effects but also economic costs [137]. From the patient's point of view, in this phase of life these unnecessary declines in quality of life (QoL) should be avoided and the transition from cancer-directed to host-directed therapies [138] should already be accomplished (Fig. 5C). Maintenance and maximization of comfort should undoubtedly be the GoC in this phase of life (Fig. 5B).

For assessment of treatment response in these late phases of metastasis or heavily pre-treated patients, we monitor cancer-directed therapies according to the respective criteria we mainly established in first-line trials, e.g. RECIST (response evaluation criteria in solid tumors) resulting in response criteria such as complete response, partial remission, stable disease or progressive disease. Alternatively, quantification of metastasis volume in the mainly affected organ(s) might be assessed (in relation to non-affected organ tissue) [139] or in the whole body, e.g. by PET/CT (positron emission computed tomography/computed tomography). As a result, growth over time in a respective organ would be recorded including tumor volume doubling time to adequately report on the dynamics of metastasis progression in the most affected organ. Together with innovative biomarkers [139–141], the use of wearables and (organ-specific) patient-reported outcomes [34, 142–144], this alternative imaging evaluation could be used to develop a multimodal classification system for symptom burden, QoL and the degree of organ destruction. This may potentially help to earlier identify the organ most at risk for organ failure, to take specific measures protecting the organ at an earlier stage, to monitor the organ-destruction more precisely, and to plan and use local therapies more effectively.

The visualization of the therapeutic indications (Fig. 5C) also illustrates very obviously that the traditional dichotomous division of GoC into curative and palliative is no longer up to date [132]. More and more oncologists are nowadays not only avoiding the traditional dichotomy, but are also adapting GoC to other chronic diseases. [145]. They additionally use the GoC prevention, prolongation of life, maintenance of organ function, maintenance of comfort, maximization of comfort, and end of life care (EoL)[146]. We have also considered this development and ongoing debate in the proposed model and have adapted the GoCs accordingly (Fig. 5B). It is necessary to note that there are fluid transitions between the GoCs [132], however in both directions under cancer-directed therapy [147]. Due to this and to the increasing number of unexpected courses of therapy, it is recommended to avoid the term palliative care [148] during cancer-directed systemic therapies. If one transfers this discussion to the bow tie model, early palliative care should be renamed into “supportive services during disease-enhanced management” [149]. This would surely increase the acceptance of this offer in earlier tumor stages among oncologists, as well as patients and their relatives [149].

Finally, being aware of all limitations and that transitions between the clinical and biological metastatic phases are fluently and these processes run in different phases parallel in various organs, we nevertheless propose the Bio Therapeutic Goals of Cancer Care Model (Fig. 5A-C), which aligns the metastatic biology, clinical stages, goals of care and therapeutic indications and which can already be used for everyday purposes as follows:

- First, define the GoC according to the tumor stage.
- Then, select the appropriate and indicated therapeutic measures.
- Use this approach during interdisciplinary tumor board meetings and shared decision making so that everyone is always aware of the common GoC.
- If the GoC is cure or prolongation of life, appropriate preventive measures (e.g. quit smoking, structured exercise programs) should be suggested.
- Keep in mind that tertiary prevention could improve QoL, progression-free survival and overall survival of cancer patients.
- Supportive therapies should cover all the needs of cancer patients from the very beginning.
- Supportive therapies should be reviewed and adjusted regularly throughout the course of the disease.
- Identify the most affected organ early on and pay particular attention to it.
- Please note that at some point during the course of metastasis, you will need to switch from cancer- to host-directed therapy.
- Each GoC has therapeutic measures (cancer- and host-directed); therefore, we never stop therapy, but merely change the GoC and the type of therapy accordingly.

## Conclusion and perspectives

As in the early days of tumor surgery in localized tumor stages, today clinicians and scientists must investigate the clinical impact of innovative systemic therapies in advanced tumor stages. In doing so, we must not ignore alternative and secondary dissemination, organ failure and cancer-related deaths. A better understanding of the metastatic organ destruction processes and their heterogeneity will be the basis for better therapy selection and monitoring in the future. This knowledge is furthermore necessary for offering personalized tumor treatments, avoiding ineffective cancer-directed therapy, preventing unnecessary side effects and identifying new therapeutic approaches in the later phases of metastasis. The Bio Therapeutic Goals of Cancer Care Model can help to communicate and explain the GoC in a more adequate way to basic, translational and clinical scientists as well as clinicians of various specialties. Finally, if we want to improve maintenance of organ function of the metastatic organ, we also need to reach much better understanding of the transitions between localized and limited macro-metastasis, extensive macro-metastasis and metastatic organ insufficiency of the affected organ.

## Data Availability

No datasets were generated or analysed during the current study.

## Abbreviations

ALL: Acute lymphatic leukemia
BAM: Border-associated macrophage
CNS: Central nervous system
CSF: Cerebrospinal fluid
CTC: Circulating tumor cell
CVL: Central vascular ligation
DTC: Disseminated tumor cell
ECC: Exfoliated cancer cell
EMP: Epithelial mesenchymal plasticity
EMT: Epithelial mesenchymal transition
EoL: End of life
GoC: Goal of care
HGP: Histological growth pattern
L1CAM: L1 cell adhesion molecule
LMM: Leptomeningeal metastasis
MAG: Myelin-associated glycoprotein
MIC: Metastasis-initiating cells
MME: Metastatic microenvironment
MMPI: Macro-metastatic parenchyma interface
NCAM: Neural cell adhesion molecule
NETs: Neutrophil extracellular traps
OMD: Oligo-metastatic disease
OSM: Organ-specific macrophage
PMD: Pachymeningeal disease
PND: Perineural dissemination
PNI: Perineural invasion
primMIC: Metastasis inducing cells from the primary tumor
QoL: Quality of life
RTM: Resident tissue macrophage
secMIC: Secondary dissemination of metastasis-inducing cells
TME: Tumor microenvironment
TVDT: Tumor volume doubling time
